# Improvement of the Centrifugal Force in Gravity Driven Method for the Fabrication of Highly Ordered and Submillimeter-Thick Colloidal Crystal

**DOI:** 10.3390/polym13050692

**Published:** 2021-02-25

**Authors:** Ting-Hui Chen, Shuan-Yu Huang, Syuan-Yi Huang, Jia-De Lin, Bing-Yau Huang, Chie-Tong Kuo

**Affiliations:** 1 Department of Physics, National Sun Yat-sen University, Kaohsiung 80424, Taiwan; final951753@gmail.com (T.-H.C.); xena343111@gmail.com (S.-Y.H.); 2 Department of Optometry, Chung Shan Medical University, Taichung 40201, Taiwan; syhuang@csmu.edu.tw; 3 Department of Opto-Electronic, National Dong Hwa University, Hualien 974301, Taiwan; jdlin1218@gms.ndhu.edu.tw; 4 Department of Optometry, Shu-Zen Junior College of Medicine and Management, Kaohsiung 82144, Taiwan

**Keywords:** Stöber process, silica particles, self-assembly, Bragg reflection, colloidal crystal

## Abstract

In this paper, we propose a modified gravity method by introducing centrifugal force to promote the stacking of silica particles and the order of formed colloidal crystals. In this method, a monodispersed silica colloidal solution is filled into empty cells and placed onto rotation arms that are designed to apply an external centrifugal force to the filled silica solution. When sample fabrication is in progress, silica particles are forced toward the edges of the cells. The number of defects in the colloidal crystal decreases and the structural order increases during this process. The highest reflectivity and structural order of a sample was obtained when the external centrifugal force was 18 G. Compared to the samples prepared using the conventional stacking method, samples fabricated with centrifugal force possess higher reflectivity and structural order. The reflectivity increases from 68% to 90%, with an increase in centrifugal force from 0 to 18 G.

## 1. Introduction

Colloidal crystals are periodic structures composed of monodispersed colloidal particles [1]. Colloidal crystals are photonic crystals that are used in optoelectronics [1,2,3], lasers [1,3], displays [4,5] and LEDs [6], etc. The optical characteristics [1,2,3] of colloidal crystals are attributed to the Bragg reflections [1,2,3] of ordered arrangements of monodispersed particles. The self-assembly of monodispersed colloidal particles is the main method for fabricating colloidal crystals [2,7,8,9,10,11,12,13,14,15,16]. There are several methods for motivating this self-assembly, including gravity-driven methods [2,8], electrophoresis [9,10,11], centrifugal-force-driven methods [12] and capillary-force-driven methods [13,14]. Among these methods, the gravity-driven method is a low-cost and simple method for fabricating colloidal crystals, as the gravitational force is always present and does not require special equipment. In the gravity-driven method, the colloidal particles in a solution are forced by gravity, and driven to stack into colloidal crystals. Colloidal particles are aligned from the bottom of the container, and slowly stacked into a compact and ordered colloidal structure.

However, it is difficult to control the thickness of photonic crystals using the gravity-driven method, as the thickness of the resulting structure is determined by the concentration, the volume of the colloidal solution and particle size [17,18]. The structural order and corresponding reflectance of photonic crystals fabricated using the gravity-driven method must be improved. An appropriate driving force from the lateral directions during the fabrication process can be beneficial in terms of developing photonic crystals with higher structural order and enhanced optical characteristics [19]. Additionally, some methods have to be implemented in large containers [20,21,22], which could result in the inefficient use of particles during the stacking process.

In our experiments, centrifugal force was introduced into a modified gravity-driven method. In the modified gravity-driven method, the thickness of photonic crystals is controlled by two spacers in a removable cell composed of the two spacers and two corresponding glass substrates. Based on the small spaces in the cell, a high-concentration solution is used in the fabrication process to form photonic crystals with large areas. A colloidal solution is injected into the glass cell, meaning the amount of colloidal solution can be controlled precisely to reduce particle waste. Centrifugal force is then applied during particle stacking. By modifying the rotational speed and radius of the rotation arm, samples can be fabricated under various centrifugal forces. We also discuss the effects of centrifugal force on the features of stacked photonic crystals by comparing photonic crystals stacked with centrifugal force to photonic crystals stacked without centrifugal force.

## 2. Materials and Methods

Monodispersed silica colloidal particles were prepared using the modified Stöber method [23,24,25]. In this process, appropriate amounts of tetraethyl orthosilicate (98%, from ACROS, Geel, Belgium), ammonia (from ECHO, Kaohsiung, Taiwan), deionized water and ethanol (from J. T. Baker, Phillipsburg, NJ, USA) were mixed and reacted for approximately 24 h, and then washed alternatingly with deionized water and ethanol for at least six rounds. The average particle size of the silica colloidal particles was approximately 210 nm.

**Figure 1:**
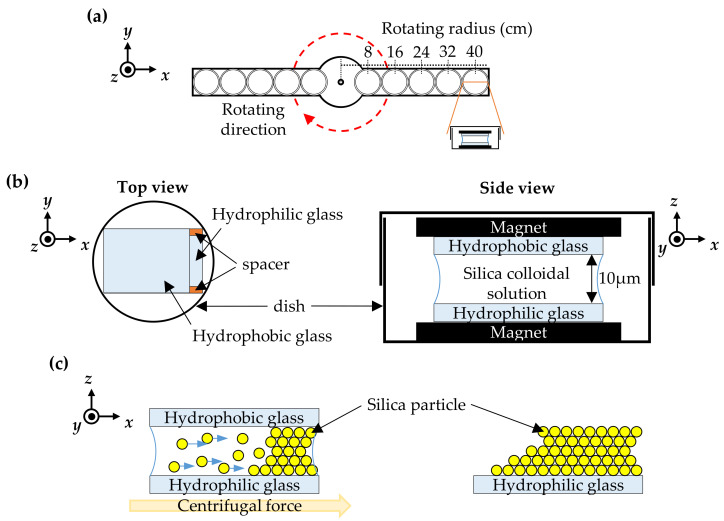
Schematics of the experimental setup for depositing and stacking silica particles with different radii of rotation and rotational speeds. (**a**) The rotation arms; (**b**) composition of the cell; and **(c)** schematic diagram of silica particles during the process.

The colloidal silica particles were deposited on a hydrophilic-treated glass plate, which was prepared by immersing a clean glass substrate into a piranha solution consisting of sulfuric acid (H_2_SO_4_, from ECHO, Kaohsiung, Taiwan) and hydrogen peroxide (H_2_O_2_, from ECHO, Kaohsiung, Taiwan), using the centrifugal method to form a colloidal crystal structure. A hydrophobic-treated glass plate, which was prepared by immersing a glass substrate in a surfactant (DTAB, dodecyl(trimethyl)ammonium bromide, from Sigma Aldrich, St. Louis, MO, USA) water solution, was placed on top of the silica droplet. Two 10-µm-thick spacers were used to form a gap between the glass plates. The two glass plates were treated to possess hydrophobic and hydrophilic properties, respectively, to allow the colloidal crystals to easily remain on the hydrophilic-treated glass plate [26]. These glass plates were fixed by magnets and placed in humid dishes that were placed at various positions (8, 16, 24, 32 and 40 cm) on the rotator, as shown in Figure 1. The positions of the samples were defined by their distances from the rotating axis.

The stacking process of the colloidal particles took place under centrifugal force for approximately 12 h around 25 °C. During this process, the particles were driven by centrifugal force, and moved horizontally outward. Three rotational speeds (150, 200 and 250 rpm) were used in the stacking process. All samples, including no rotated stacked sample, were prepared at room temperature for 12 h, and with no vibrations. After the stacking process was completed, the hydrophobic-treated glass plates were removed and the deposited particles remained on the hydrophilic-treated glass plate substrates.

A spectrometer (UV4000, from Ocean optics, Orlando, FL, USA) was used to measure the reflection spectra of the colloidal crystals. A white light source (wavelength: 400 to 900 nm) was used for the reflection measurements. Light from the light source irradiated the samples, and the reflected light was received by a fiber and delivered to the spectrometer. Because the particles were highly ordered, Bragg reflections occurred, resulting in high reflectance at a specific wavelength. Reflection efficiency depends on the order of the stacked structures. Cracks and defects in the stacked structure would increase the scattering of light, and lead to a decrease in the reflectance of the stacked structures [27]. A thermal field-emission scanning electron microscope (SEM, FEI: inspect F50) was used to visualize the colloidal crystal structures. The acceleration voltage of the SEM was set to 10 kV. Because silica is non-conductive, the samples were pre-coated with a layer of 5-nm-thick platinum to enhance electrical conductivity before capturing SEM images. The silica colloidal crystal structures presented in this work have a face-centered cubic (FCC) structure [2,16,28,29,30], and the diffraction surface is the (111) plane [7,16] parallel to the glass substrate.

## 3. Results and Discussion

**Figure 2:**
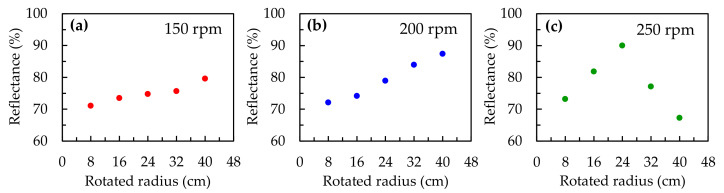
Variation in the reflectance values of samples stacked at different positions with rotational speeds of (**a**) 150 rpm; (**b**) 200 rpm; and (**c**) 250 rpm.

The measured reflectance values of silica colloidal crystals stacked at different rotational speeds (150, 200, and 250 rpm) and radii of rotation (8, 16, 24, 32, and 40 cm) are presented in Figure 2. The concentration of the colloidal silica solution was 30 wt %. According to the previous literature [7,16] and SEM images, the reflective plane of the colloidal crystals in our reflection measurements is (111). The lattice constants of the colloidal crystals can be estimated using the following equation

(1) $$d\;=\sqrt{\frac{2}{3}}a,$$

where *d* is the lattice constant of the silica colloidal crystal and *a* is the diameter of a single colloidal particle. The central wavelength of the Bragg reflection of the sample is defined as follows

*Mλ* = 2*nd*cos*θ*, (2)

where *m* is the order of the Bragg reflection, *λ* is the Bragg reflective wavelength of the colloidal crystal, *n* is the effective refractive index of the colloidal crystal, *θ* is the angle between the incident beam and the normal direction of the sample and *d* is the lattice constant of the colloidal crystal. In our experiments, because the incident beams travelled along the normal directions of the samples, and *m* was equal to one, this formula can be simplified as:

*Λ* = 2*nd*. (3)

The theoretical reflective wavelength of a sample can be calculated as 475 nm for our experiments, as the effective refractive index of the colloidal crystals is 1.33 and the lattice constant is 178.6 nm.

Figure 2a presents the variation in the reflectance values of samples stacked at different positions, but with a fixed rotational speed of 150 rpm. The reflectance increases from 71.1% to 79.6% with an increase in the radius of the rotation arm from 8 to 40 cm. Similar to Figure 2a, the reflectance values of the samples produced at 200 rpm also increase from 72.2% to 87.4%, with an increase in the radius of the rotation arm, as shown in Figure 2b. Figure 2c presents the results at 250 rpm. One can clearly see that the reflectance values of the samples are 73.2%, 81.8%, 90%, 77.2% and 67.3%, when the radius of the rotation arm is 8, 16, 24, 32 and 40 cm, respectively. As the radius of the rotation arm increases, the reflectance of the sample first increases, and then decreases.

**Figure 3:**
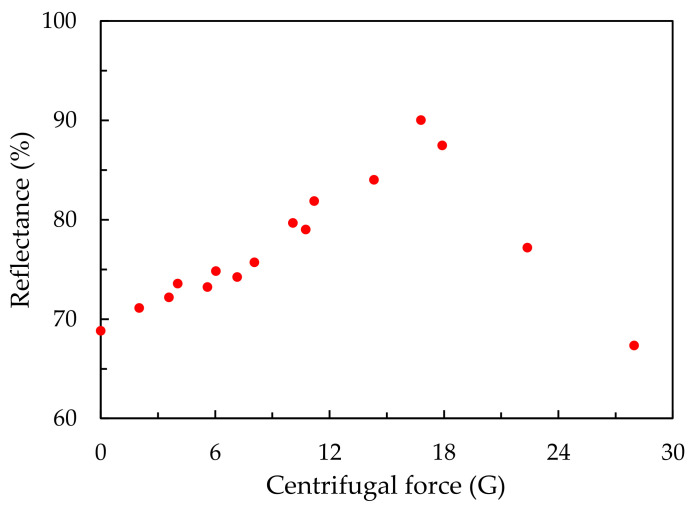
Reflectance values of samples stacked with a 30 wt % silica colloidal solution under different centrifugal forces.

To determine the relationship between the reflectance, radius of the rotation arm and rotational speed in our experiments, the centrifugal forces on the samples during the stacking process were calculated using the formula for uniform circular, as shown in Figure 3. The unit of centrifugal force used in Figure 3 is gravitational acceleration (G). Additionally, the reflectance of a sample fabricated without centrifugal force was also measured, and is plotted in Figure 3 for comparison. The centrifugal forces in our experiments were in the range of 0–28 G. The reflectance value of the sample stacked with no centrifugal force is 68%. The reflectance of the samples increases from 68% to 90%, with an increase in centrifugal force from 0 to 18 G. The highest reflectance of a sample was measured at 24 cm and 250 rpm. If there is no centrifugal force during the stacking process, particles are only driven by gravity and stacked on the hydrophilic-treated glass plate. The centrifugal force in our experiments not only provided a stronger driving force than gravity, but also moved the particles outward toward the edge of the cell. A stronger driving force stacks particles more quickly, and with fewer voids.

**Figure 4:**
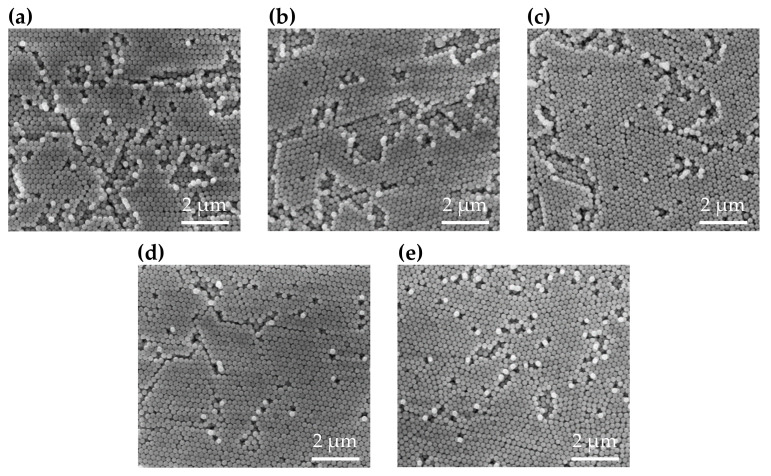
SEM images of a structure stacked with a 30 wt % silica colloidal solution and under centrifugal forces of (**a**) 0; (**b**) 7; (**c**) 14; (**d**) 18; and (**e**) 21 G.

SEM images are presented in Figure 4. In the existing literature, the structures of deposited silica have been reported as FCC [2,16,28,29,30]. We can confirm the effects of our method by observing the lattice areas in SEM images. The order of the lattice structures of the samples increases as the centrifugal force increases from 0 to 18 G, and decreases when the centrifugal force is 21 G. The SEM images also show that the optimal structure of the silica deposit appears at approximately 18 G. The SEM images demonstrate that the voids in the colloidal crystals stacked with high centrifugal force are smaller than those in the crystals stacked with low centrifugal force when the centrifugal force does not exceed 18 G. When the centrifugal force is greater than 18 G, deformations begin to appear in the stacked structures. The silica particles in the cell are mainly forced to align by centrifugal force, as the force of gravity (1 G) is smaller than the centrifugal force. When the centrifugal force is below 18 G, it is not sufficient for the silica particles to fill all cells, meaning voids are left in the colloidal crystals by the convection of particles on the substrate [31]. Figure 4a–d demonstrate that with a higher centrifugal force acting on the samples during the stacking process, fewer voids are generated in the resulting structure. The structural order increases based on the reduction of defeats in the structures. When the centrifugal force increases, silica particles are more effective at filling and stacking into an organized structure. However, if the force is greater than 18 G, the structure exhibits deformations, as shown in Figure 4e. These deformations break the uniformity of the colloidal crystal, thereby reducing the structural order and reflectance of the sample.

**Figure 5:**
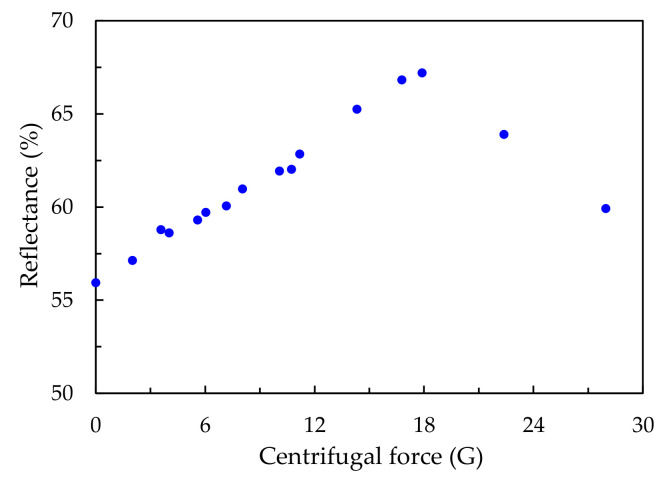
Reflectance values of samples stacked with a 50 wt % silica colloidal solution under different centrifugal forces.

To determine the effects of the concentration of the solution on the formed colloidal crystals, silica particle deposits stacked with a 50 wt % silica colloidal solution were also examined. The results are presented in Figure 5. One can see that the reflectance increases from 56% to 67% when the centrifugal force increases from 0 to 18 G, whereas the reflectance decreases when the centrifugal force is greater than 18 G. Compared to the samples stacked with a 30 wt % solution, the reflectance values of the samples stacked with a 50 wt % solution are lower by 10% to 20%.

**Figure 6:**
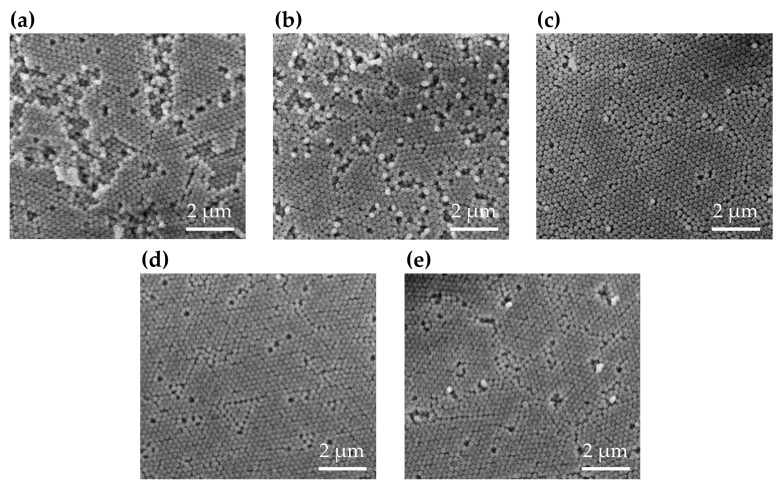
Scanning electron microscope (SEM) images of structures stacked with a 50 wt % silica colloidal solution under centrifugal forces of (**a**) 0; (**b**) 7; (**c**) 14; (**d**) 18; and (**e**) 21 G.

SEM images of the structures stacked with the 50 wt % silica colloidal solution also reveal that voids decrease when the centrifugal force increases to 18 G, and deformations appear when the centrifugal force is greater than 18 G, as shown in Figure 6. The lattice size of the silica deposits is smaller than that of the samples stacked with a 30 wt % silica solution; this may be a result of the high silica concentration. First, the colloidal silica particles form small bundles. When the stacking process occurs, the small bundles of silica particles approach each other, and then integrate into large domains. Because the distance between particles in the 50 wt % solution is relatively small, this solution has less space for particles to adjust their positions to integrate into a uniform domain. Therefore, the final lattice size is smaller than that of the samples stacked with the 30 wt % silica solution.

**Figure 7:**
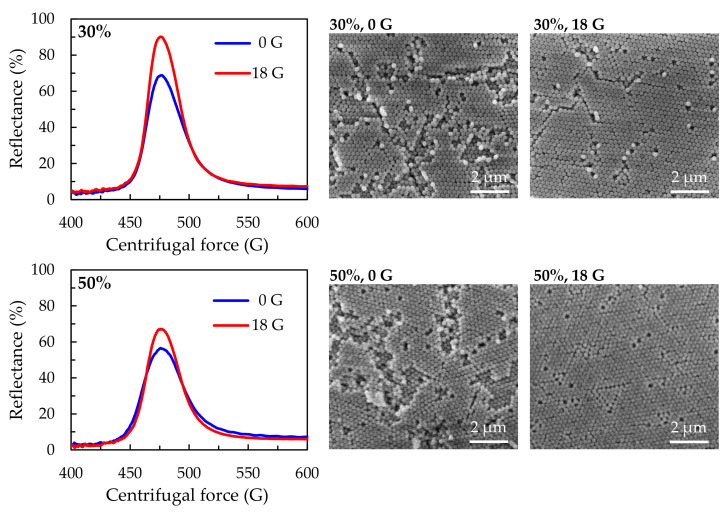
Reflection spectra and SEM images of samples stacked at 0 and 18 G.

The results presented above demonstrate that the non-rotated samples possess relatively low reflectance and structural order. This implies that centrifugal force can improve the stacking of silica particles. The reflection spectra and SEM images of the samples fabricated under centrifugal forces of 0 G and 18 G are presented in Figure 7. In Figure 7, one can see that whether the concentration of the solution in the stacking process is 30 wt % or 50 wt %, the reflectance and the structure of the resulting sample are improved by centrifugal force. In the 30 wt % case, the reflectance increases from 68% to 90% under an external centrifugal force of 18 G. In the 50 wt % case, the reflectance increases from 56 to 67 wt % under a centrifugal force of 18 G. The increased reflectance of the samples fabricated under centrifugal force stems from the higher orders of the resulting photonic structures, which are verified by the corresponding SEM images.

## 4. Conclusions

In this study, we demonstrated that centrifugal force can significantly improve the assembly of particles when using the modified gravity method. Monodispersed silica colloidal solutions were filled into cells consisting of two spacers and two glass substrates. The samples were placed on rotation arms to apply centrifugal force to the samples. Under this centrifugal force, the silica particles in the samples were forced outward. The voids in the structures are reduced during this process, and the structural order increases. By increasing the centrifugal force acting on a sample, the ratio of the centrifugal force to the gravity acting on the sample increases, and the direction of the net force changes from downward to outward. The reflectance of colloidal crystals stacked with a 30 wt % silica solution can be improved from 68% to 90%, with an increase in centrifugal force from 0 to 18 G. When the force exceeds 18 G, the reflectance of the sample decreases as a result of structural deformation. Similarly, the reflectance of colloidal crystals stacked with a 50 wt % silica solution also improved from 56% to 67%. The highest reflectance and structural order among the samples were obtained when the centrifugal force was 18 G. Compared to samples fabricated using the conventional method with no rotation, the samples fabricated with centrifugal force exhibited higher reflectance and structural order.
